# Compound Dihuang Granule Inhibits Nigrostriatal Pathway Apoptosis in Parkinson’s Disease by Suppressing the JNK/AP-1 Pathway

**DOI:** 10.3389/fphar.2021.621359

**Published:** 2021-04-08

**Authors:** Li Wang, Yu-fang Yang, Long Chen, Zhu-qing He, Dian-yong Bi, Lei Zhang, Yan-wu Xu, Jian-cheng He

**Affiliations:** ^1^ Department of Diagnostics of Traditional Chinese Medicine, School of Basic Medicine, Shanghai University of Traditional Chinese Medicine, Shanghai, China; ^2^ Experiment Center, Shanghai Municipal Hospital of Traditional Chinese Medicine, Shanghai University of Traditional Chinese Medicine, Shanghai, China; ^3^ Experiment Center for Science and Technology, Shanghai University of Traditional Chinese Medicine, Shanghai, China; ^4^ Department of Biochemistry, School of Basic Medicine, Shanghai University of Traditional Chinese Medicine, Shanghai, China

**Keywords:** Compound Dihuang Granule, Parkinson’s disease, apoptosis, traditional Chinese medicine, JNK/AP-1 pathway, network pharmacology

## Abstract

Compound Dihuang Granule (CDG) is widely used in traditional Chinese medicine (TCM) for the treatment of Parkinson’s disease (PD). It has been shown to alleviate PD symptoms. However, the molecular mechanisms of its action have not been established. To establish the molecular mechanisms of CDG against PD, we used TCM network pharmacology methods to predict its molecular targets and signaling pathways, followed by experimental validation. The Core Protein protein interaction (PPI) network of the 150 intersections between CDG and PD-related genes, comprising 23 proteins, including CASP3 (caspase-3), MAPK8 (JNK), FOS (c-Fos), and JUN (c-Jun). KEGG and GO analyses revealed that apoptotic regulation and MAPK signaling pathways were significantly enriched. Since c-Jun and c-Fos are AP-1 subunits, an important downstream JNK effector, we investigated if the JNK/AP-1 pathway influences CDG against apoptosis through the nigrostriatal pathways in PD rat models. Molecular docking analysis found that the top three bioactive compounds exhibiting the highest Degree Centrality following online database and LC-MS analysis had high affinities for JNK. Experimental validation analysis showed that CDG decreased the number of rotating laps and suppressed the levels of phosphorylated c-Jun, c-Fos, and JNK, as well as the number of TUNEL positive cells and the cleaved caspase-3 level in the nigrostriatal pathway. Furthermore, CDG treatment elevated the number of TH neurons, TH expression level, and Bcl-2/Bax protein ratio in a 6-OHDA-induced PD rat. These findings are in tandem with those obtained using SP600125, a specific JNK inhibitor. In conclusion, CDG suppresses the apoptosis of the nigrostriatal pathway and relieves PD symptoms by suppressing the JNK/AP-1 signaling pathway.

## Graphical Abstract

**Figure:**
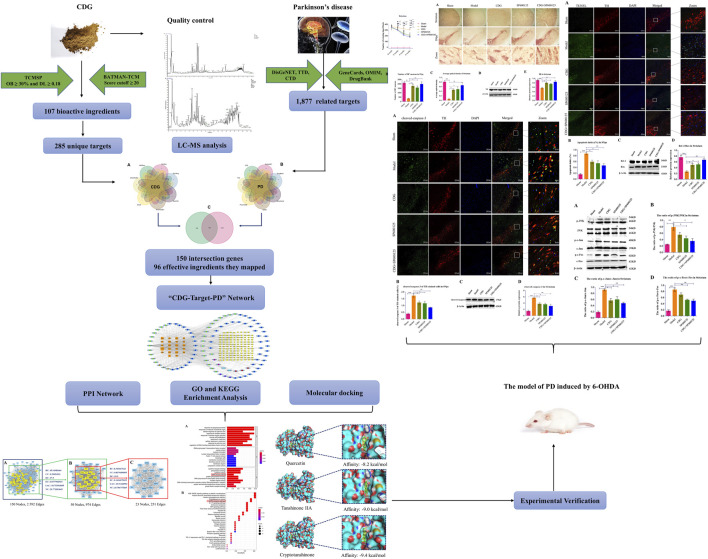
The graphical abstract of this study.

## Introduction

Globally, Parkinson’s disease (PD) is the second most prevalent neurodisease. It is estimated that up to 2% of people aged >65 years are affected by PD (GBD 2016 Neurology Collaborators, 2019). PD is characterized by bradykinesia, rigidity, rest tremor, and gait disturbance (Lees, et al., 2018). It often results in mental disorders, including changed cognitive functions and mental state abnormalities (Kehagia, 2016). The pathogenesis of PD is initiated by dopaminergic (DA) neuronal degeneration in the substantia nigra pars compacta (SNpc), leading to reduced striatum DA levels (Ernst et al., 2014). The etiology of PD is complex and is influenced by social, environmental, genetic, and biological factors. It has been documented that DA neuron apoptosis due to oxidative stress (Chi et al., 2019) or mitochondrial dysfunctions (Lin, et al., 2019) are the major mediators of caspase-3 activation (Hanrott et al., 2006). Although PD symptoms can be clinically managed, there are no effective therapies for this disease (Esteves, 2017). Single-target drugs have often been found to be ineffective. Therefore, multitargeted TCMs with substantial pharmacological activity could be potential alternatives with good clinical effects. According to the TCM theory, most PD patients were “liver and kidney deficit,” “viscera dysfunction,” “blood stasis,” “phlegm,” “wind,” and “poison” mutual knot. Compound Dihuang Granule (CDG) could enrich yin in the liver and kidneys, calming the liver and inhibiting yang, promoting blood circulation, removing blood stasis, detoxifying the body and removing phlegm, and exhibiting significant clinical benefits (Wang and He, 2019; Hu et al., 2019; Liang and He, 2018a; Liang and He, 2018b). The results of a previous clinical trial showed that CDG decoction combined with Madopar in the treatment of PD could improve the symptoms, reduce adverse action rate, and have significant synergistic effect on Madopar. The total effective rates of the “CDG+ Madopar” group and the “Madopar” group were 60.00 and 24.00% (*p* > 0.01), respectively. The patients’ heart, liver, and kidney function of the intervention group had no significant changes before and after treatment (Xu et al., 2019). CDG is made up of seven traditional Chinese medicines: shú dì huáng (*Rehmannia glutinosa* (Gaertn.) DC.), bái sháo (*Paeonia lactiflora* Pall), gōu téng (Uncis *Uncaria rhynchophylla* (Miq.) Miq. ex Havil), Zhēn zhū mǔ (*Hyriopsis cumingii* (Lea)), Dān shēn (*Salvia miltiorrhiza* Bunge), Shī chāng pǔ (*Acorus tatarinowii* Schott), and Quán xié (*Buthus martensii* Karsch). We have previously shown that CDG suppresses the apoptosis of PD (Hu et al., 2019). Studies have established that some herbal compounds are protective in PD models (Tseng et al., 2012; Liang et al., 2016). However, given that TCM has multiple components and target features, it is challenging to fully establish the mechanisms involved in CDG effects against PD. Moreover, its antiapoptotic effects have not yet been elucidated.

Network pharmacology is an emerging discipline in systems biology (Zhou et al., 2019) that has been used to assess the pharmacological value of medicines with multiple compounds (Zhang Y.-F. et al., 2019). Furthermore, it is often used to determine the molecular basis of complex chronic disorders, such as neurodegenerative and cardiocerebrovascular pathologies (Wang et al., 2019). Therefore, network analysis using various existing pathologies has elucidated the mechanisms of multitarget drug action in complex diseases. In this study, we used LC-MS to analyze the bioactive components of CDG for quality control and then used network pharmacology to predict the mechanisms underlying CDG action against PD. Then, we experimentally tested the potential mechanism of CDG in PD. The graphical abstract of this study is shown in GRAPHICAL ABSTRACT.

## Materials

Male Sprague-Dawley rats, weighing 160–200 g, were obtained from the animal experimental center, Shanghai University of TCM (license No. SYXK (Hu) 2020-0009). The test Chinese medicines were purchased from Shanghai Leiyunshang Pharmaceutical Co., Ltd. 6-Hydroxydopamine (6-OHDA) and apomorphine (APO) were purchased from Sigma Chemicals. Antibodies against c-Fos, cleaved caspase-3 (Asp175), p-JNK (phosphor-Thr183/Tyr185), JNK, Bcl-2, Bax, and tyrosine hydroxylase (TH) were bought from Cell Signaling Technology. Antibodies against p-c-Fos (phosphor-Ser362), p-c-Jun, and c-Jun (phosphor-Ser73) were bought from Abcam. Anti p-c-Fos (phosphor-Ser362) was purchased from Invitrogen. SP600125 (S1066) was purchased from Selleck. *In situ* cell death detection kit was purchased from Roche Molecular Systems. VECTASTAIN® Elite ABC Kit (mouse IgG), VECTASTAIN® Elite ABC Kit (Rabbit IgG), and DAB substrate were bought from Vector Laboratories. The BCA kit was purchased from Thermo Fisher Scientific. All chemicals were of analytical grade and were commercially available.

### CDG Preparation and Administration

CDG (lot number: 20140102) was prepared by Shanghai Traditional Chinese Medicine Pharmaceutical Technology Co., Ltd. (Table1). Briefly, the herbs were sliced and soaked (shú dì huáng 20 g, bái sháo 30 g, gōu téng 15 g, Zhēn zhū mǔ 15 g, Dān shēn 20 g, Shī chāng pǔ 12 g, and Quán xié 2 g) in distilled water for 30 min. They were then boiled twice (1 h each), first in 10 and then in 8 volumes of distilled water. The extracts were pooled and filtered before being concentrated into a cream at a relative density of 1.3. The preparation was dried, sifted, and powdered into CDG. The CDG standard used in this study conformed to the Chinese pharmacopeia (2010 version). The quality control analysis of CDG was performed using LC-MS (Supplementary File S1). Based on the human-rat dose-conversion formula [DB (rat) = DA (human)*7/388], 6.3-fold the normal dose for an adult human was determined as the appropriate dose for the rat models, which were intragastrically given 7 g/kg/day CDG or an equal volume of distilled water.

**TABLE 1:** Components of Compound Dihuang Granule (CDG).

Chinese name	Pharmacopeia	Common name	Voucher numbers
Shu-Di-Huang	*Rehmannia glutinosa* (Gaertn.) DC.	Rehmanniae radix praeparata	CDG01–130306
Bai-Shao	*Paeonia lactiflora* Pall	Paeoniae radix alba	CDG02-DH2012071703
Gou-Teng	Uncis *Uncaria rhynchophylla* (Miq.) Miq. ex Havil	Uncariae ramulus cum uncis	CDG03-HY2012102204
Zhen-Zhu-Mu	*Hyriopsis cumingii* (Lea)	Margaritifera concha	CDG04-HY2012040501
	*Pteria martensii* (Dunker)		
Dan-Shen	*Salvia miltiorrhiza* Bge	Salviae miltiorrhizae radix et rhizoma	CDG05-YT2012091506
Shi-Chang-Pu	*Acorus tatarinowii* Schott	Acori tatarinowii rhizome	CDG06-LY2012080321
Quan-Xie	*Buthus martensii* Karsch	Scorpio	CDG07-YT2012092411

The ratio of these herbs was 20:30:15:15:20:12:2.

### Network Pharmacology Analysis of CDG

#### Prediction of Potential CDG Targets in PD Therapy

The pharmacological information of CDG was retrieved from http://bionet.ncpsb.org/batman-tcm/ (Liu et al., 2016) and http://tcmspw.com/(Ru et al., 2014) databases. The components of each herb with a drug-likeness (DL) ≥0.18 or score cutoff ≥20 and an oral bioavailability (OB) ≥30% were selected as bioactive ingredients while their target genes, as cataloged in the databases, were identified as CDG targets.

Disease related genes were obtained from Therapeutic Target Database (TTD, http://db.idrblab.net/ttd/), Online Mendelian Inheritance in Man (OMIM, https://omim.org/), Genecards (https://www.genecards.org/), Comparative Toxicogenomics Database (CTD, http://ctdbase.org/), DisGeNET (https://www.disgenet.org/), PharmGBK (https://www.pharmgkb.org/), and DrugBank (https://go.drugbank.com/) and combined into a list of PD-related genes. Intersection genes were then identified and considered as potential therapeutic targets. CDG components identified by these intersection targets were identified as being effective against PD.

#### Construction of Drug-Target-Disease Network

To determine the interactions between intersection targets and the CDG’s active components, we used the STRING database (https://string-db.org/) (Szklarczyk, 2018) to generate a PPI network. The data were then exported to the Cytoscape (3.7.2) to design a “Drug-Target-Disease” network.

#### PPI Network Construction and Core PPI Network Extraction

To identify the key CDG targets, a two-step screening procedure was performed to extract the core PPI network from the primary PPI network in STRING. Greater than the topological parameter medians of Eigenvector (EC), Degree (DC), Closeness (CC), Betweenness (BC), LAC, and Network (NC) were set as screen parameters.

#### Pathway and Functional Enrichment Analysis

GO and KEGG enrichment analysis of CDG’s core potential therapeutic targets were performed using the bioconductor packages (org.Hs.eg.db, colorspace, stringi, DOSE, clusterProfile, pathview, ggplot2, and limma et al.) of R (3.6.0) software. Statistical significance was set at *q* ≤ 0.05.

#### Molecular Docking

The Autodock Vina (Trott and Olson, 2010) and optimal models visualized using PyMOL (2.0) software were used to perform the molecular docking of active components.

### Experimental Validation

#### Ethical Statement

The male Sprague-Dawley rats (weighing between 160 and 200 g) used in this study were reared in the laboratory center of Shanghai University of TCM. All procedures involving rats were ethically approved (license No. SYXK (Hu) 2020-0009). Animals were kept in wire cages at a humidity of 60–65%, a temperature of 23 ± 2°C, and 12 h dark/light cycles, with free access to food and water.

#### Generation of 6-OHDA-Induced Rat PD Model

Rats were intraperitoneally anesthetized using 50 mg/kg 3% pentobarbital sodium (WS20130112) and fixed on a stereotaxic apparatus (RM2016). 6-OHDA in normal saline containing 0.2% ascorbic acid (MKBP0832V) or sterile saline (3 μl at each point) was injected at two points in the SNpc (first point at 5.2 mm posterior to the bregma, 1.0 mm right lateral to the midline, 9.0 mm below the dura mater, and second point at 5.2 mm posterior to the bregma, 2.5 mm right lateral to the midline, 8.5 mm below the dura mater) at 1 μl/min using a 5 μl Hamilton syringe. After injection, the needle was retained in the SNpc for 5 min before withdrawal at 1 mm/min. Rats were returned to the same environment they were before surgery. Two weeks after the operation, APO induced rotation tests were performed. Rats with a rotating frequency >7 turns/min were included in the PD model (Carman et al., 1991).

#### Treatments

Rats in the CDG group were intragastrically given 7 g/kg/d CDG (1 ml/100 g), and 10% DMSO was injected intraperitoneally. Rats in the SP600125 group were intraperitoneally given 30 mg/kg SP600125 solution (dissolved in 10% DMSO) and gavaged with 1 ml/100 g of saline. Rats in the CDG + SP600125 group were intraperitoneally given 30 mg/kg SP600125 immediately after gavage with 7 g/kg/d CDG. Rats in the sham group and Model group were gavaged with 1 ml/100 g of saline and intraperitoneally given 10% DMSO solution. The intraperitoneal injection volume was 0.1 ml/100 g, twice weekly for 6 weeks.

#### Rotation Test

After surgery, the contralateral rotational behavior was induced by intraperitoneally injecting rats with 0.5 mg/kg of APO. The number of rotations in the test were scored from a video recorded at weeks 0, 2, 4, and 6 for 30 min each.

#### Western Blot Analysis

Proteins were extracted from the striatum tissues using the Tissue Protein Extraction Reagent (T-PER™, Thermo Scientific) that comprised phosphatase and protease inhibitors. They were centrifuged at 4°C, for 10 min, at 12,000 g, and supernatants collected. Protein extracts were quantified using a BCA kit and denatured by heating at 95°C. 40 μg of each sample was then resolved in 10% SDS-PAGE and subsequently transferred onto a 0.45 µm PVDF membrane (EMD Millipore). Nonspecific antibody interactions were prevented by blocking the membrane with 3% BSA in TBS (Sigma-Aldrich) for 2 h at room temperature (RT). Proteins were further incubated with the following primary antibodies at the indicated concentrations: anti-TH (1:1,000), anti-Bax (1:200), anti-Bcl-2 (1:500), anti-JNK (1:100), anti-p-JNK (1:100), anti-p-c-Jun (1:1,000), anti-cleaved caspase-3 (1:1,000), anti-c-Jun (1:1,000), anti-p-c-Fos (1:1,000), and anti-c-Fos (1:1,000), overnight at 4°C. After incubation, they were washed thrice using 0.1% TBST and incubated with HRP-conjugated secondary antibodies. As loading controls, the membranes were incubated with mouse anti-β-Actin (1:3,000; Santa Cruz Biotechnology) and mouse anti-GAPDH monoclonal antibody (1:3,000; Proteintech Group) for 1 h at RT. They were then washed thrice, and the signal was developed using ECL for 1 min and then imaged on Li-cor ODDSEY (CLx-1259). Signal quantification was done on ImageJ.

#### Immunohistochemistry and Immunofluorescence

Brains specimens were sliced into coronal sections (20 μm thick) using a frozen microtome (Leica). The coronal sections were suspended in a cryoprotectant solution and kept at −20°C for histological analysis. For immunohistochemistry, similar frozen sections from each group were selected, and antigen retrieval was done using citrate buffer (0.1 M, pH 6.0), at 95°C, for 10 min. Sections were then rinsed thrice using 0.2% PBST for 10 min, permeabilized with 0.5% triton X-100 for 10 min, and blocked using 5% BSA for 1 h at RT. For immunohistochemistry, endogenous peroxidase was inactivated using 3% H_2_O_2_ for 10 min. Then, they were incubated overnight with anti-TH (Abcam, ab112) at 1:1,000 in 0.5% PBST/1% sheep serum at 37°C for 2 h, 4°C. For immunofluorescence, samples were probed with Alexa Fluor 555 goat anti-rabbit and goat anti-mouse secondary antibody, as well as with Alexa Fluor 488 (Invitrogen, at 1:1,000 dilution) for 1 h. Imaging was then done using a confocal microscope (Leica TCS SP2). For immunohistochemistry, the RP-conjugated secondary antibody (Abcam, Cambridge, MA, United States) was incubated with samples for 1 h after which the signals were developed using 3,3′-diaminobenzidine (DAB) for 2–3 min. They were then mounted on neutral balsam and observed under an Olympus BA51 photomicroscope. Obtained images were analyzed using the Pro Plus 6.0 software (Media Cybernetics).

#### TUNEL Assay

The TUNEL assay was done on brain sections following manufacturer instructions with minor modifications. Briefly, the TUNEL test was performed using the 20 μm thick frozen sections as well as an *in situ* cell death detection kit (Roche Molecular Systems, Switzerland Basel, Germany) and imaged by confocal microscopy (Leica TCS SP2, Solms, Germany). The level of cell apoptosis was determined on cells stained with DAPI (for nuclei staining) using the Image Pro Plus software (version 6.0, Media Cybernetics).

#### Statistical Analysis

Data are presented as mean ± SEM. Multiple data groups were analyzed using one-way ANOVA. Rotation test data were analyzed by multivariate analysis of variance (ANOVA) and by repeated measures of the general linear model. Data were considered significant at *p* ≤ 0.05.

## Results

### Construction of a “CDG-Target-PD” Network

**FIGURE 1:**
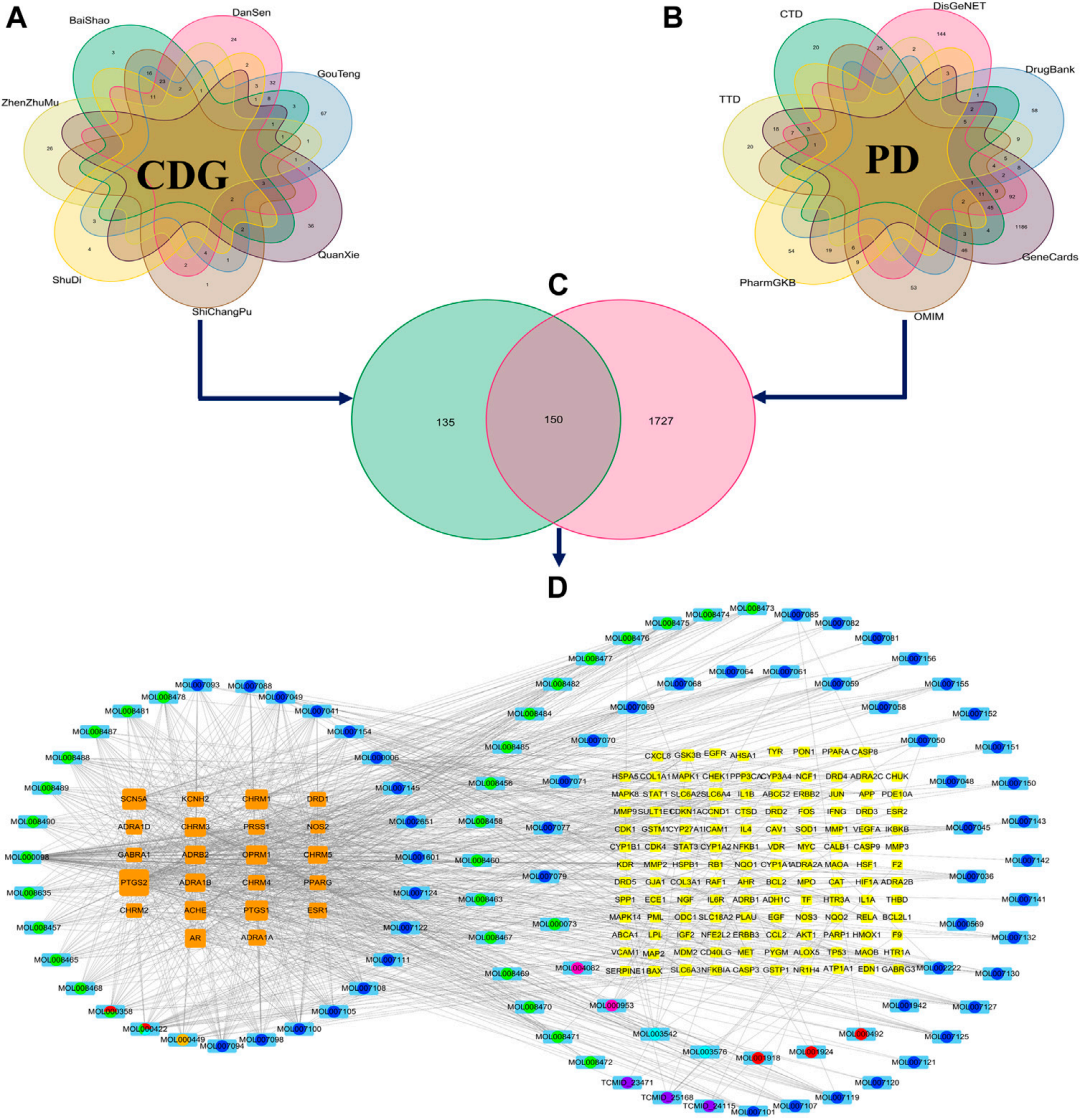
The construction of the ingredient-target network of CDG. **(A)** The collection of the targets of CDG. **(B)** The collection of the related genes of PD. **(C)** The intersection of CDG targets and PD-related genes. **(D)** The ingredient-target network of CDG. The yellow and orange squares represent targets. Big size represents the larger Degree Centrality; the colored circle with sky blue square background represents the ingredients from different herbs. Circles with different color sectors represent the ingredients from multiherbs.

A total of 107 bioactive compounds (Supplementary File S2) and their 285 nonredundant CDG targets (Supplementary File S3) were retrieved from the BATMAN-TCM and TCMSP data repositories (Figure 1A). In total, 1,877 PD-related genes were identified from disease databases (Figure 1B; Supplementary File S4). The 150 genes identified at the intersection between the two sets were considered anti-PD CDG targets (Figure 1C) while the 96 active compounds they revealed were regarded as effective CDG components. A “CDG-Targets-PD” network was constructed using STRING and Cytoscape (Figure 1D; Supplementary File S5). The top-10 compounds with the highest degree centrality (DC) were *quercetin*, *kaempferol*, *luteolin*, *tanshinone IIA*, *yohimbine*, *salviolone*, *beta-sitosterol*, *4-methylenemiltirone*, *dihydrotanshinlactone*, and *2-isopropyl-8-methylphenanthrene-3,4-dione*.

### PPI Network Construction and Core PPI Network Extraction

**FIGURE 2:**
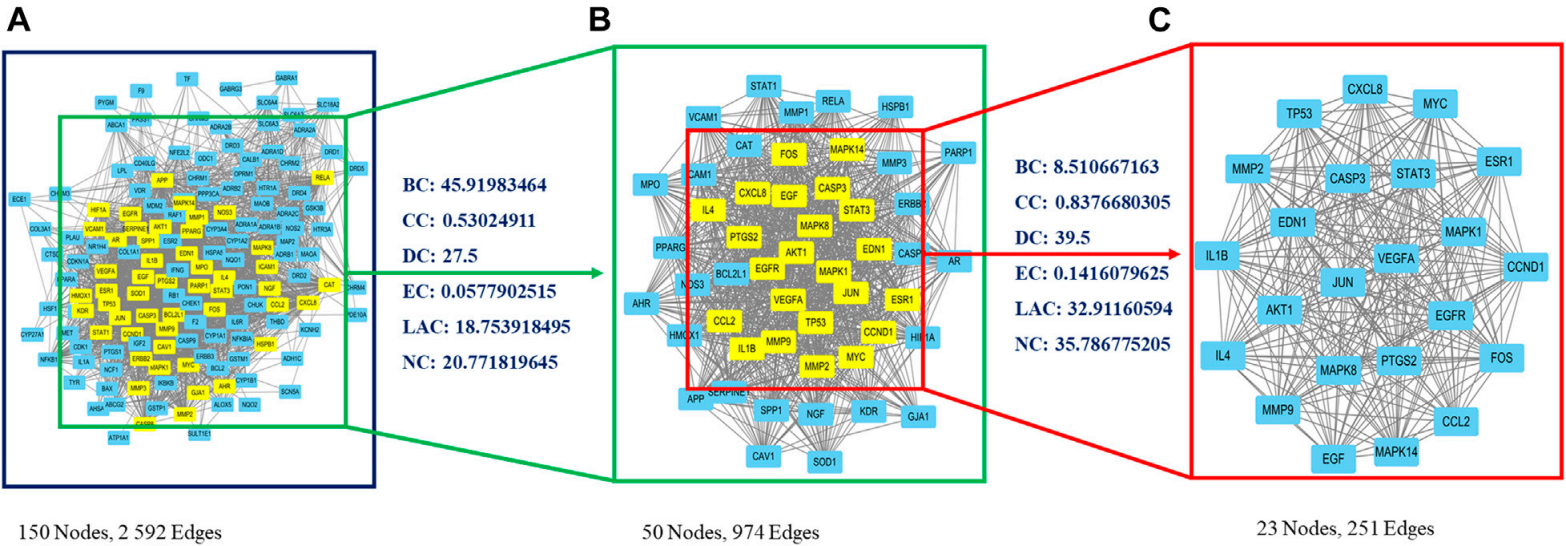
Identification of core candidate targets of CDG against PD. **(A)** The interactive PPI network of CDG putative targets and PD-related targets. **(B)** PPI network of significant proteins extracted from **(A)**. **(C)** Core PPI network of candidate CDG targets for PD treatment extracted from **(B)**. BC, betweenness centrality; CC, closeness centrality; DC, degree centrality; EC, eigenvector centrality; LAC, local average connectivity-based method; NC, network centrality.

A PPI network was created using the STRING website, and resulting data were imported into Cytoscape. The primary network had 150 nodes and 2,592 edges (Figure 2A). Next, CytoNCA plugin was used to calculate topological parameters and to extract the core PPI network in two steps. The core PPI network contains 23 nodes and 251 edges. CASP3 (caspase-3), MAPK8 (JNK), FOS (c-Fos), and JUN (c-Jun) were identified in the core network (Figure 2C; Supplementary File S6).

### KEGG and GO Enrichment Analyses

**FIGURE 3:**
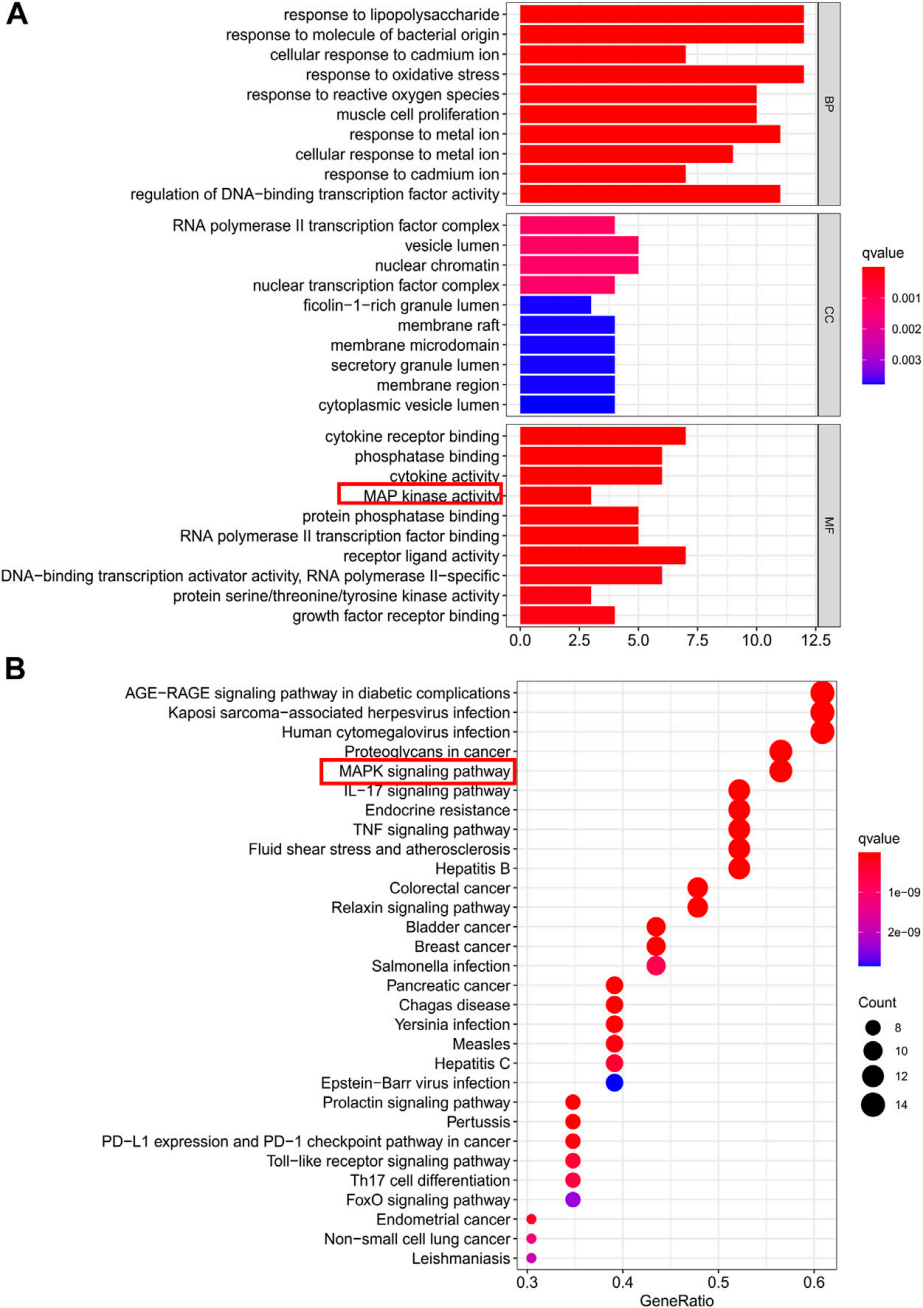
Gene ontology terms and KEGG pathway enrichment of core candidate targets of CDG against PD. **(A)** GO of core candidate targets of CDG against PD. The top-10 terms in each GO category with *p* Adjust Value <0.05 were selected. BP, biological process; CC, cellular components; MF, molecular function. **(B)** KEGG pathway enrichment of core candidate targets of CDG against PD. The top-30 pathways that had significant changes of P Adjust Value <0.05 were identified.

GO term enrichment analysis of the 23 core PPI network genes was used to determine the biological functions of CDG against PD. In total, 91, 2, and 1,766, significant terms were found for molecular function (MF), biological process (BP), and cellular component (CC), respectively. The top-10 significantly enriched terms in each category are shown in Figure 3A. Regulation of the execution phase of apoptosis and cellular component disassembly in the execution phase of apoptosis were both enriched (Supplementary File S7).

KEGG pathway analysis of the 23 core PPI network genes identified 136 significantly enriched signaling pathways, including IL-17, TNF, MAPK, and apoptotic signaling pathways (Supplementary File S8). The top-30 significantly enriched terms are shown in Figure 3B.

### Molecular Docking

**FIGURE 4:**
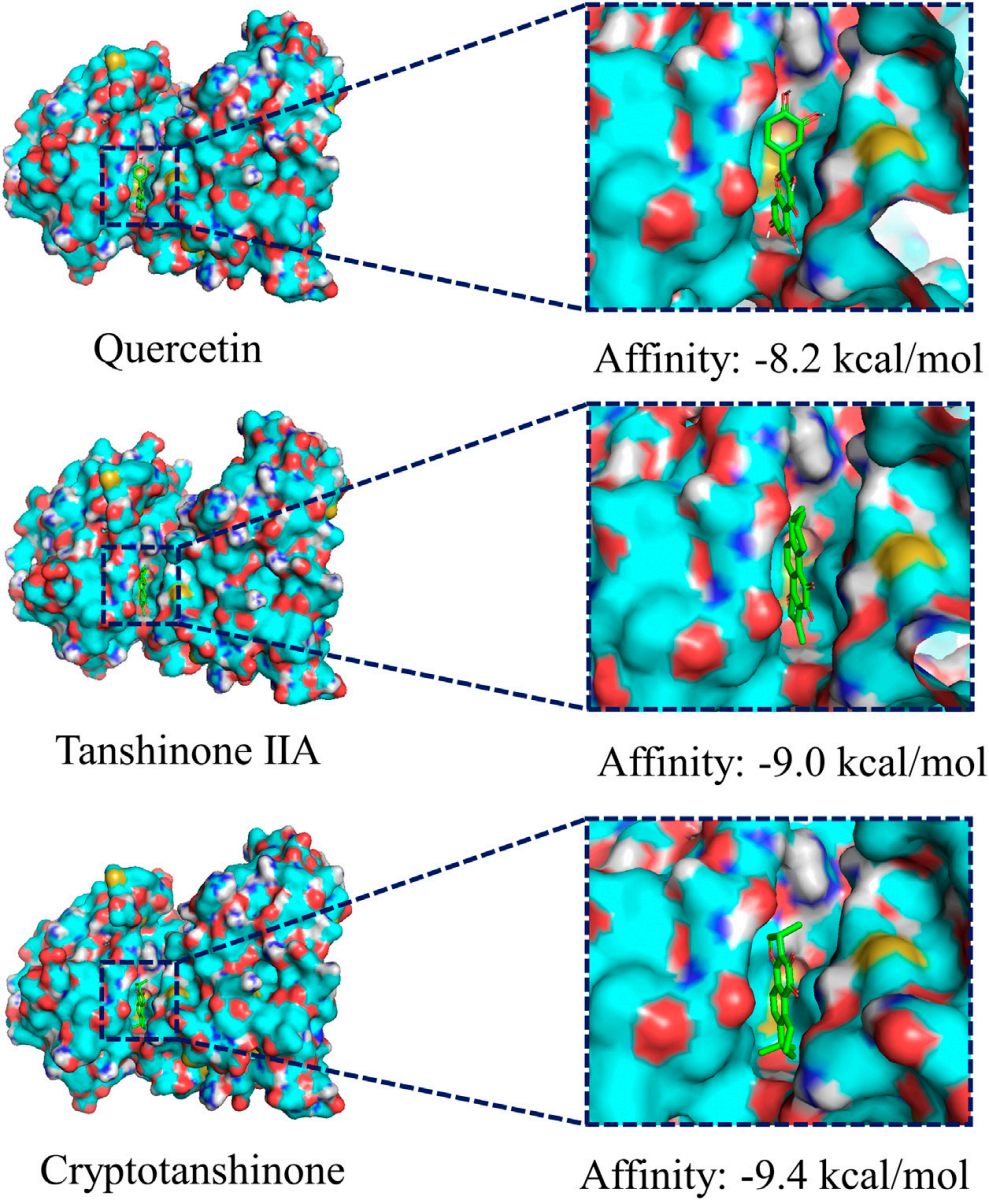
Molecular docking of the ingredients with MAPKS (JNK). The top three ingredients with the largest DC from both the database and the results of LC-MS analysis. The ligands (ingredient molecules) are shown in stick mode. The receptor is shown in surface model.

We chose the top three compounds (*quercetin*, *tanshinone IIA*, and *cryptotanshinone*) with the highest DC in the “CDG-Targets-PD” network, which were also identified by LC-MS for molecular docking analysis with JNK and their optimal docking and affinity models were derived (Figure 4). The affinities of *quercetin*, *tanshinone IIA*, and *cryptotanshinone* for JNK were −8.0 kcal/mol, −9.0 kcal/mol, and −9.4 kcal/mol, respectively, implying a high affinity. These data suggest that CDG exerts its anti-PD effects by influencing apoptosis. The MAPK signaling pathway may be the mechanism mediating this effect. Since FOS and JUN are subunits of the transcription factor, AP-1, a critical downstream JNK effector, we determined if the JNK/AP-1 pathway, an important branch of MAPK signaling, contributes to the CDG’s anti-PD effects by influencing apoptosis in PD rat models.

### Experimental Validation

#### CDG Alleviates Motor Deficits in 6-OHDA-Induced PD in Rats

**FIGURE 5:**
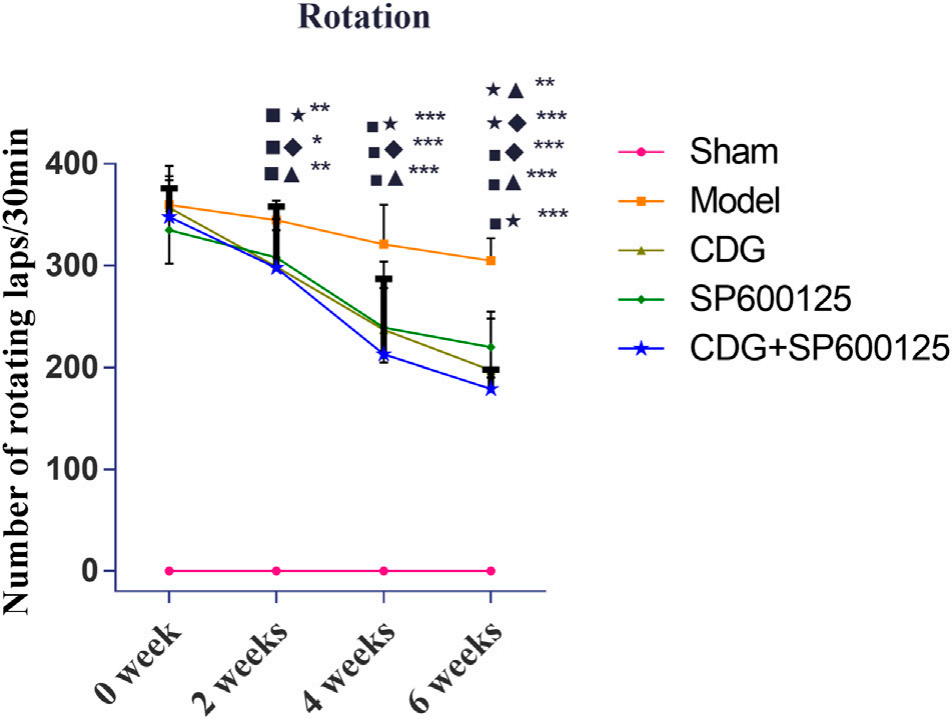
Comparison of the number of revolutions of each group at 0, 2, 4, and 6 weeks. Statistical analysis was performed with repeated measures and multivariate analysis of variance (ANOVA), *n* = 9. Significant differences were indicated by *p < 0.05; **p < 0.01; ***p < 0.001.

Motor deficit analysis revealed that there was no rotation behavior in control rats (Figure 5). At week 0, 6-OHDA-induced rat models exhibited irritable and violent rotation behavior, with the number of rotations significantly higher relative to the sham group (*p* < 0.001), indicating successful establishment of the PD model. Consistently, the number of rotations by CDG, SP600125, and CDG + SP600125 treated rats was not significantly different on week 0. However, at weeks 2, 4, and 6, the number of rotations by the CDG, SP600125, and CDG + SP600125 treatment groups significantly decreased relative to the PD model group (*p* < 0.05, *p* < 0.01). However, the number of rotations by the CDG + SP600125 group was significantly lower than that of the CDG and SP600125 groups at week 6 (*p* < 0.01). At other timepoints, there were no statistical differences between intervention groups (*p* > 0.05).

#### Treatment With CDG Prevented the Loss of Nigrostriatal DA in PD Rats

**FIGURE 6:**
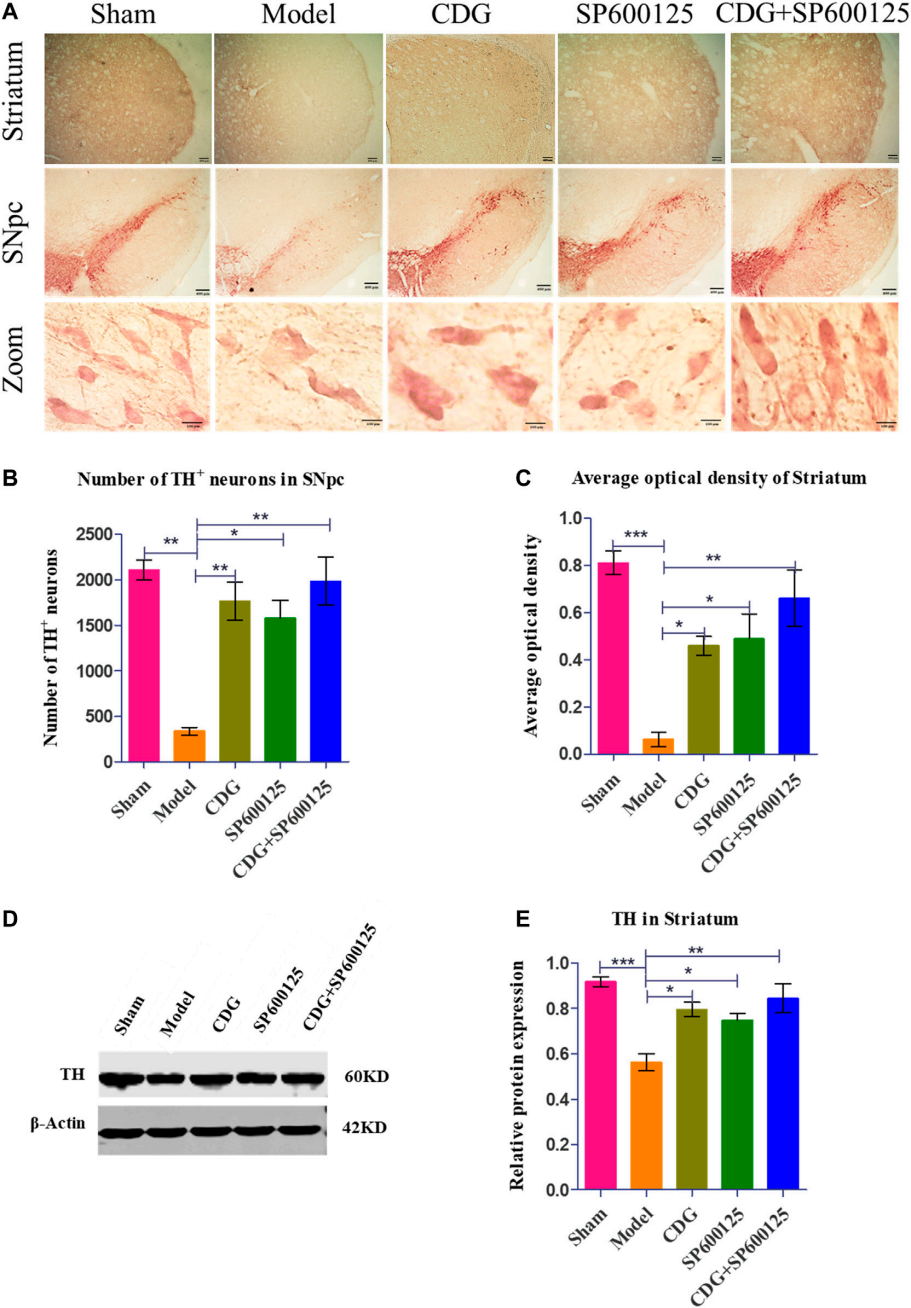
6-OHDA-induced loss of DA neurons in the nigrostriatal pathway of the rat brain. **(A)** DAB staining of TH on midbrain sections in each group (Scale bar: 400 µm; Zoomed Scale bar: 100 µm). **(B)** Stereological counts of TH-positive cells of the SNpc at 6 weeks after 6-OFDA intoxication. **(C)** Average optical density of striatum in each group. **(D)** The expression level of TH proteins was detected with Western Blot in the striatum. **(E)** The expression level of TH protein in each group. β-Actin served as control. Statistical analysis was performed with One-Way ANOVA, *n* = 3. Significant differences were indicated by *p < 0.05, **p < 0.01, ***p < 0.001.

DAB staining revealed that TH neurons in the SNpc of the sham group were brown, with plump bodies, high numbers, and clear outline and processes. Neuronal atrophy was observed in rats of the PD model group, with vague contours and protrusions. Compared to the sham group, the number of TH neurons in the PD model group was significantly low (*p* < 0.01). At week 6 of intervention, compared to the model group, CDG was shown to significantly increase the number of TH cells (*p* < 0.01). Moreover, CDG enhanced the average optical density of the striatum of PD rats when compared to the model group (*p* < 0.05). Treatment with SP600125 and CDG + SP600125 exhibited consistent findings with those of CDG (*p* < 0.05, *p* < 0.01, Figures 6A–C). Western blot analysis of TH protein levels showed consistent results (*p* < 0.01 or *p* < 0.05, Figures 6D,E). This indicated that CDG, SP600125, and CDG + SP600125 treatment attenuated 6-OHDA-stimulated DA neuronal injury by suppressing neuron loss and elevating TH expression.

#### CDG Reduced 6-OHDA-Induced Apoptosis in the Nigrostriatal Pathway of PD Rats

**FIGURE 7:**
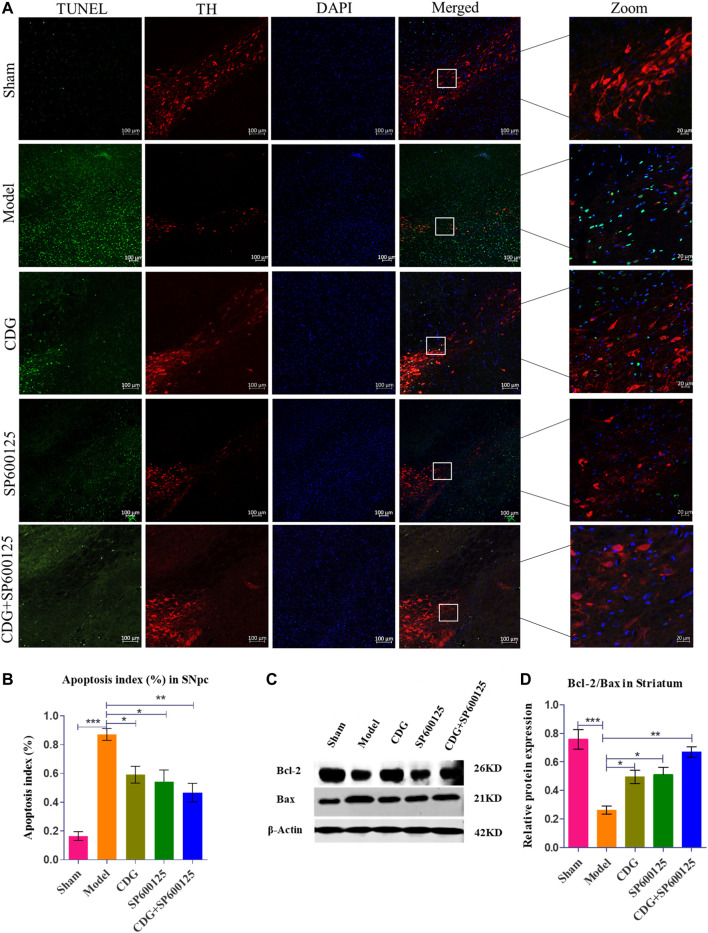
6-OHDA induced nigrostriatal pathway apoptosis of the PD rat brain. **(A)** TUNEL assay of apoptosis in each group. Representative confocal fluorescent images of the SNpc with TUNEL (green), TH (red), and DAPI (blue) (scale bar = 100 µm; Zoomed Scale bar; 20 µm). **(B)** Apoptosis index of the SNpc at 6 weeks after 6-OHDA intoxication. **(C,D)** The protein expression level of Bcl-2 and Bax were detected with Western Blot in the striatum. Statistical analysis was performed with One-Way ANOVA, *n* = 3. Significant differences were indicated by *p < 0.05, **p < 0.01, ***p < 0.001.

TUNEL analysis of SNpc apoptosis in each group was examined relative to TH expression in the SNpc. Compared to the sham group, TUNEL positive cells were significantly elevated in the model group (*p* < 0.001, Figures 7A,B). Treatment with CDG, SP600125, or CDG + SP600125 significantly reduced the number of TUNEL positive cells in the SNpc when compared to the model group (*p* < 0.05 or *p* < 0.01). Western blotting analysis revealed a marked reduction in the ratio of Bcl-2/Bax in the striatum of the 6-OHDA-lesioned group when compared to the sham group (*p* < 0.001). Compared to the model group, CDG, SP600125, and CDG + SP600125 treatment significantly elevated the ratio of Bcl-2/Bax (*p* < 0.05, *p* < 0.01). These findings imply that CDG, SP600125, and CDG + SP600125 suppress apoptosis in 6-OHDA-induced PD rats.

#### CDG Suppressed Neuronal Cleaved Caspase-3 Levels

**FIGURE 8:**
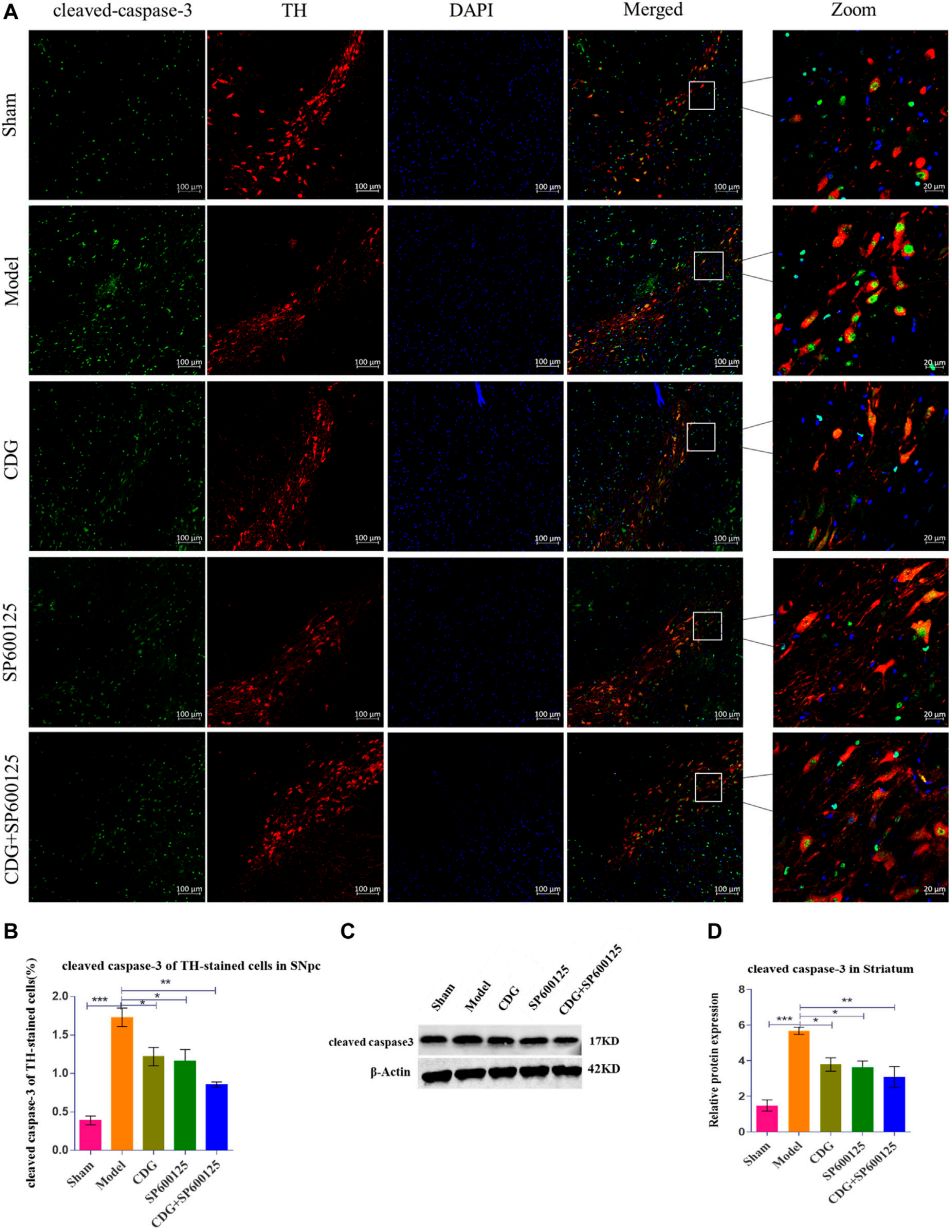
Expression of cleaved caspase-3 protein. **(A)** cleaved caspase-3 was detected by IF in each group. cleaved caspase-3 (green), TH (red) and DAPI (blue). (scale bar = 100 µm; Zoomed Scale bar: 20 µm). **(B)** The cleaved caspase-3 of TH-stained cells were calculated. **(C,D)** The expression level of cleaved caspase-3 protein was detected with Western Blot in the Striatum. β-Actin served as control. Statistical analysis was performed with One-Way ANOVA, Turkey’s multiple comparison test, *post hoc*, *n* = 3. Significant differences were indicated by *p < 0.05, **p < 0.01, ***p < 0.001.

Cleaved caspase-3 levels were examined by immunofluorescence and costandardized to TH expression in rat SNpc (Figures 8A,B). The number of apoptotic neurons in PD rat model was found to be significantly higher when compared to the sham group (*p* < 0.001). After 6 weeks of treatment, CDG was shown to significantly reduce the number of apoptotic neurons compared to the model group (*p* < 0.05). Similar results were obtained upon SP600125 or CDG + SP600125 treatment for 6 weeks (*p* < 0.05, *p* < 0.01). Notably, protein levels of cleaved caspase-3 were significantly high in PD rat models compared to the sham group (*p* < 0.001). After 6 weeks of treatment, CDG significantly suppressed cleaved caspase-3 levels when compared to the model group (*p* < 0.05). Moreover, SP600125 and CDG + SP600125 significantly suppressed cleaved caspase-3 protein levels (*p* < 0.05, *p* < 0.01, Figures 8C,D).

#### CDG Suppressed JNK/AP-1 Activity in the SNpc-Striatum Axis of PD Rats

**FIGURE 9:**
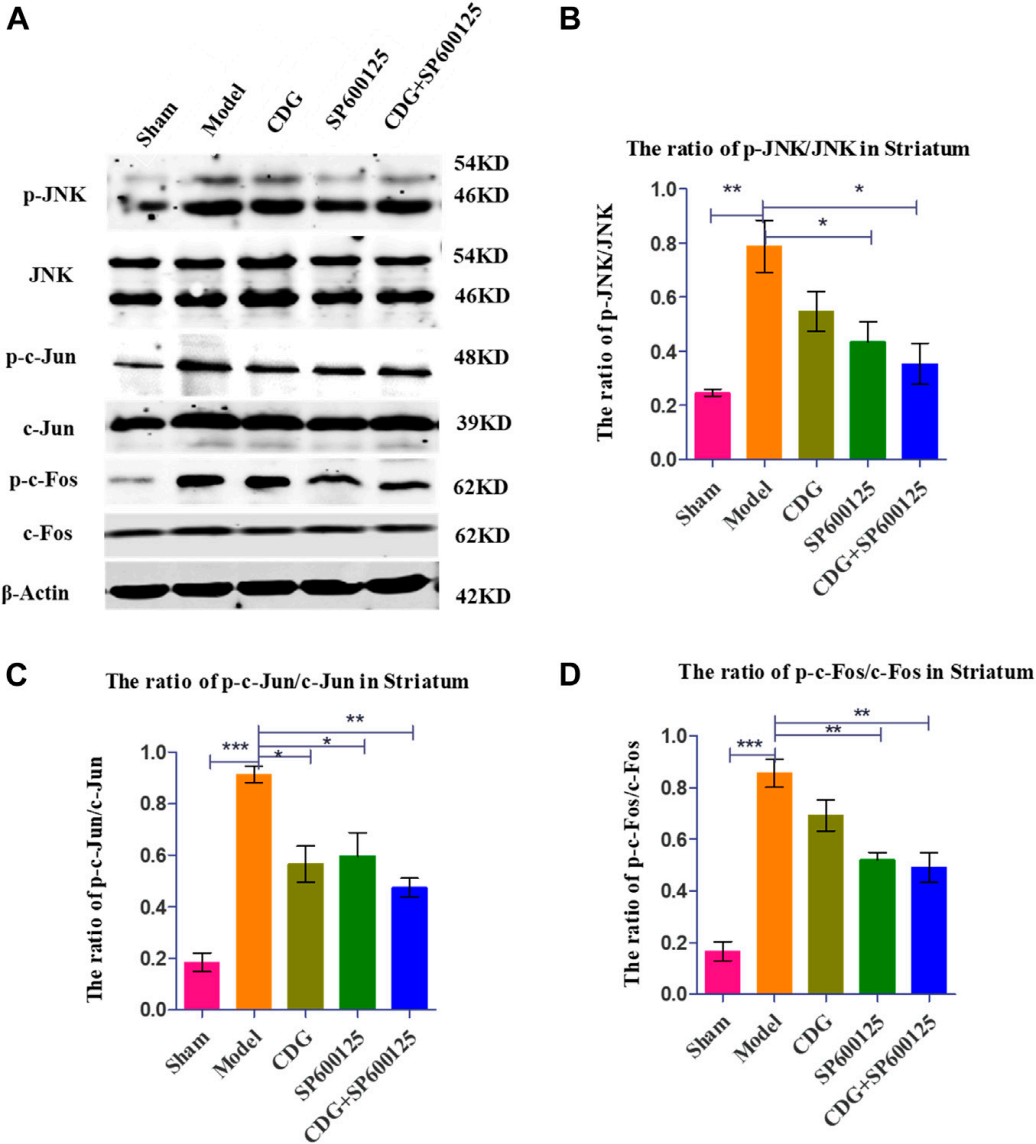
Inhibition of related protein expression in the JNK/AP-1 signaling pathway. **(A)** The expression levels of p-JNK, JNK, p-c-Jun, c-Jun, p-c-Foc, and c-Fos were detected with Western Blot in the striatum. **(B–D)** The expression levels of p-JNK/JNK, p-c-Jun/c-Jun were calculated. GAPDH served as control. Statistical analysis was performed with One-Way ANOVA, Turkey’s multiple comparison test, post hoc, *n* = 3. Significant differences were indicated by *p < 0.05, **p < 0.01, ***p < 0.001.

Western blot analysis of striatum tissue revealed significantly higher ratios of p-JNK/JNK, p-c-Jun/Jun, and p-c-Fos/Fos protein levels in PD rats compared to controls (*p* < 0.001, Figures 9A–D). CDG treatment inhibited p-JNK/JNK, p-c-Jun/Jun, and p-c-Fos/Fos levels (*p* < 0.05 or *p* < 0.01). Similar results were obtained upon treatment with SP600125, a specific JNK inhibitor (Figures 9A–D). Treatment with CDG + SP600125 significantly suppressed the activation of key JNK/AP1 pathway factors compared to either treatment alone.

## Discussion

PD is a progressive neurodegenerative disease whose global prevalence is rising. Due to its complex underlying mechanisms, single-target drugs are therapeutically less effective. The multicomponent, multitarget action of TCMs exhibits diverse therapeutic effects through the synergistic activity of various pharmacologically active compounds (Li C. et al., 2017). Therefore, treating PD with TCM may be a good choice. However, inferring the mechanisms by which TCM acts is challenging. In this study, CDG, a frequently used TCM, was analyzed using a combination of network pharmacologic analysis and experimental validation to evaluate its mechanism/s of action in inhibiting DA neuronal apoptosis in PD rats. We screened its active ingredients and identified its nonredundant targets in BATMAN-TCM and TCMSP databases. PD-related genes were found in the GeneCards, OMIM, CTD, TTD, DisGeNET, PharmGBK, and DrugBank. Our data revealed 150 genes at the intersection of the two sets, and the 96 active compounds they identified were considered effective CDG components against PD. Next, a “CDG-Targets-PD” network was established and the top-10 compounds with the highest DC were identified as *quercetin*, *kaempferol*, *luteolin*, *tanshinone IIA*, *yohimbine*, *salviolone*, *beta-sitosterol*, *4-methylenemiltirone*, *dihydrotanshinlactone*, and *2-isopropyl-8-methylphenanthrene-3,4-dione*. PPI network analysis revealed that caspase-3 (CASP3), MAPK8 (JNK), and two AP1 subunits, FOS and JUN, were components of the core PPI network. GO and KEGG pathway analyses identified MAPK signaling and apoptosis to be significantly enriched. These data suggest that CDG exerts its anti-PD therapeutic effects by modulating apoptosis through MAPK signaling.

CDG composition analysis using LC-MS identified 115 compounds in *Rehmannia glutinosa* (Gaertn.) DC., *Paeonia lactiflora* Pall, Uncis *Uncaria rhynchophylla* (Miq.) Miq. ex Havil, *Hyriopsis cumingii* (Lea), *Salvia miltiorrhiza* Bunge*, Acorus tatarinowii* Schott*,* and *Buthus martensii* Karsch*.* Many of these components have previously been reported to have therapeutic effects in PD models. *Quercetin*, an active component of Uncis *Uncaria rhynchophylla* (Miq.) Miq. ex Havil, has been reported to ameliorate motor deficits, protect against DA neurodegeneration, and reduce 6-OHDA-induced neurotoxicity and to be an anti-inflammatory agent (Ay et al., 2017; Tamtaji et al., 2020). *Tanshinone IIA*, a major component of *Salvia miltiorrhiza* Bge, has been shown to elevate TH protein levels and suppress caspase-3 expression in SNpc, thereby protecting DA neurons (Tian et al., 2018). It has been shown that *cryptotanshinone*, from *Salvia miltiorrhiza* Bge, significantly suppresses apoptosis by restoring mitochondrial functions in PD patient-derived hiNPCs (Lee et al., 2018). The other identified active ingredients include *paeoniflorin*, *isorhynchophylline* and *verbascoside,* which were from *Paeonia lactiflora* Pall, Uncis *Uncaria rhynchophylla* (Miq.) Miq. ex Havil, and prepared *Rehmannia glutinosa* (Gaertn.) DC. Paeoniflorin has been reported to reduce MPTP-induced DA cell loss in a dose-dependent manner (Zheng et al., 2017). Moreover, *isorhynchophylline* was found to inhibit the generation of oxidative stress and apoptotic cell death in 1-methyl-4-phenylpyridinium (MPP^+^) induced PC12 cells (Li et al., 2017). *Verbascoside* elevates TH-positive cells in PD (Liang and He, 2018). The results of our “CDG-Targets-PD” network analysis were used to perform molecular docking analysis for the top-3 components with the highest DC (*quercetin, tanshinone IIA,* and *cryptotanshinone*), which were identified in the online pharmacological database and by LC-MS, with JNK. The strong affinities of *quercetin* (−8.2 kcal/mol), *tanshinone IIA* (−9.0 kcal/mol), and *cryptotanshinone* (−9.4 kcal/mol) for JNK were all below −7.0 kcal/mol, suggesting that JNK is a CDG target. These findings confirm our postulation that CDG components have synergistic therapeutic effects. To test this possibility, we investigated the therapeutic effects of CDG on a PD rat model that was established by the injection of unilateral stereotactic of 6-OHDA into the SNpc, a classic *in vivo* model of PD pathogenesis. The animals were treated with 7 g/kg/d CDG. Following induction with APO, the PD rat models exhibited a clear rotation behavior. The comparison between groups treated with 6-OHDA at week 0, after successful PD model establishment, was good. At 6 weeks of treatment, CDG-treated rats exhibited significantly fewer rotation circles when compared to the untreated controls. In addition, the number of neurons, TH protein expression level, and average optical density of the striatum in the CDG-treated group increased significantly. Similar results were obtained upon treatment with SP600125, a specific JNK inhibitor, indicating that CDG exerts its anti-PD effects by inhibiting the JNK pathway.

Bcl-2 inhibits apoptosis and enhances survival, while Bax enhances apoptosis (Narita et al., 1998). Caspase-3 is a proapoptotic gene. In its activated form, cleaved caspase-3 mediates apoptosis. Suppressed caspase-3 activation indirectly indicates suppressed apoptosis, which may suppress PD development through neuroprotection (Liu et al., 2013; Sharifi et al., 2015). Caspase-3 knockout enhances the anti-MPTP neurotoxic effects in mice (Yamada et al., 2010). Evaluation of the mechanisms underlying elevated DA neurons and ameliorated motor functions in PD rats upon CDG treatment using various approaches found that CDG reduced TUNEL positive cells, increased the ratio of Bcl-2/Bax, reduced cleaved caspase-3 positive cells, and suppressed SNpc protein expression. Similar results were obtained by JNK inhibition, suggesting that CDG suppresses apoptosis in the nigrostriatal pathway of PD rats through JNK inhibition.

Mitogen-activated protein kinase (MAPK) signal transduction pathways (signaling pathway) are important transmitters of transduction signals from cell surface receptors to nuclear internal targets and are significant pathways in eukaryotic signal transmission networks (Wei et al., 2020). There are three major MAPK signaling pathways, namely, signal-regulated kinase (ERK), c-Jun N-terminal kinase (JNK), and p38 MAPK pathways (Zhang et al., 2014). JNK activation controls diverse cellular functions such as cell proliferation, aging, and apoptosis (Xu et al., 2015). The JNK expression is closely related with neuronal apoptosis, which can be suppressed by inhibiting the activation of JNK (Pei et al., 2016). Prolonged JNK activation has been implicated in exacerbating disease phenotypes in PD models (Maitra et al., 2019). Network pharmacological analysis and experimental studies revealed that the JNK/AP-1 signaling pathway mediates apoptosis in PD. Apoptosis inhibition may ameliorate DA neuron loss in PD (Shishido et al., 2018). The activation of JNK signaling has been shown to promote the apoptosis of dopaminergic neurons in the SNpc of PD rats (Borsello and Forloni, 2007; Zhang et al., 2019). JNK and c-Jun phosphorylation are crucial apoptosis drivers (Spigolon et al., 2018). Once activated, they can associate with members of c-Fos and c-Jun family to form AP-1. C-Jun/AP-1 activation during MPP^+^ induced cell death in striatum has been reported (Yang et al., 2010). Studies have documented that JNK inhibitors are neuroprotective against PD (Huang et al., 2018; Chambers et al., 2011). SP600125, a specific inhibitor of JNK signaling, inhibits the MPTP-activated JNK/c-Jun pathway in DA neurons and suppresses cox-2, thereby protecting DA neurons. SP600125 also protects N-type neuroblastoma SH-SY5Y from MPP^+^ induced cell death (Yang et al., 2010). Since CASP3, MAPK8 (JNK), FOS, and JUN are core PPI components of CDG network targets, we examined whether the JNK/AP-1 pathway mediates the effects of CDG on apoptosis in PD rat models. Thus, SP600125 was also selected as the positive control in this study.

*In vivo* analysis revealed that the ratios of p-c-Fos/c-Fos, p-JNK/JNK, and p-c-Jun/p-c-Jun in the PD model group were significantly higher compared to the sham group and that apoptosis was significantly increased. Compared to the model group, CDG treatment significantly reduced the ratio of p-JNK/JNK, p-c-Fos/c-Fos, and p-c-Jun/p-c-Jun, while suppressing apoptosis. Similar results were obtained with SP600125 treatment, implying that CDG suppresses apoptosis in SNpc of PD rat models by suppressing the activation of key JNK/AP1 pathway factors. Therefore, CDG exerts its antiapoptotic effects in PD by suppressing JNK/AP1 signaling.

TCM treatments, including CDG, are characterized by the synergistic effects of their various pharmacologically active compounds and multiple targets and pathways, a conclusion that is supported by pharmacologic network and molecular docking analyses. *In vivo* results confirmed that the therapeutic effect of CDG + SP600125 was better than that of CDG or SP600125 alone, implying synergistic effects by multiple CDG active components on JNK/AP1 signaling. Its effects on other pathways have not yet been established.

## Conclusion

**FIGURE 10:**
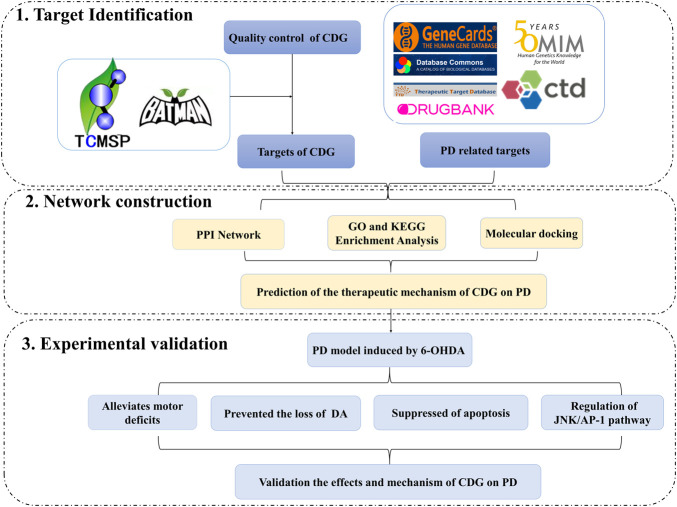
A flowchart of the research design for investing the therapeutic mechanisms of Compound Dihuang Granule (CDG) on PD by integrating target identification, network analysis, and experimental validation.

In this study, we investigated the molecular mechanisms involved in the CDG improvement of motor deficits and enhanced DA expression in neurons of PD rat models using an integrated strategy of network pharmacological analysis and experimental validation. PPI network analysis revealed CASP3, MAPK8, FOS, and JUN as core targets of the network. GO and KEGG analyses revealed MAPK signaling and apoptosis to be the significantly enriched pathways. Based on these predictions, experimental analysis confirmed that, to exert its therapeutic effect, CDG improves motor deficits, enhances the number of DA neurons, and suppresses the apoptosis of the SNpc through the JNK/AP-1 signaling pathway. Similar results were obtained when using SP600125, a JNK inhibitor. As a TCM compound, CDG has the basic characteristics of multiple pathways and multiple targets. Based on the results of CDG + SP600125 treatment, we postulated that CDG may also act on other pathways. KEGG enrichment results also showed that the HIF-1 signal pathway, the JAK-STAT signal pathway, necroptosis, autophagy, and mitophagy were all significantly enriched. Whether they also mediate the antagonistic effect of CDG against PD is the limitation of this study, and these mechanisms need to be further verified by experiments. Nevertheless, the combination of network pharmacological prediction and experimental verification effectively unveiled the molecular basis of the therapeutic effect of CDG against PD (Figures 10). This provides insights for related research and practice and also informs the rational use of CDG in clinical practice.

## Data Availability

The original contributions presented in the study are included in the article/Supplementary Material; further inquiries can be directed to the corresponding authors.
