# Corrigendum: Compound Dihuang Granule inhibits nigrostriatal pathway apoptosis in Parkinson’s disease by suppressing the JNK/AP-1 pathway

**DOI:** 10.3389/fphar.2023.1320322

**Published:** 2023-12-20

**Authors:** Li Wang, Yu-fang Yang, Long Chen, Zhu-qing He, Dian-yong Bi, Lei Zhang, Yan-wu Xu, Jian-cheng He

**Affiliations:** ^1^Department of Diagnostics of Traditional Chinese Medicine, School of Basic Medicine, Shanghai University of Traditional Chinese Medicine, Shanghai, China; ^2^Experiment Center, Shanghai Municipal Hospital of Traditional Chinese Medicine, Shanghai University of Traditional Chinese, Shanghai, China; ^3^Experiment Center for Science and Technology, Shanghai University of Traditional Chinese Medicine, Shanghai, China; ^4^Department of Biochemistry, School of Basic Medicine, Shanghai University of Traditional Chinese Medicine, Shanghai, China

**Keywords:** compound Dihuang Granule, Parkinson’s disease, apoptosis, traditional Chinese medicine, JNK/AP-1 pathway, network pharmacology

## Abstract

**Figure:**
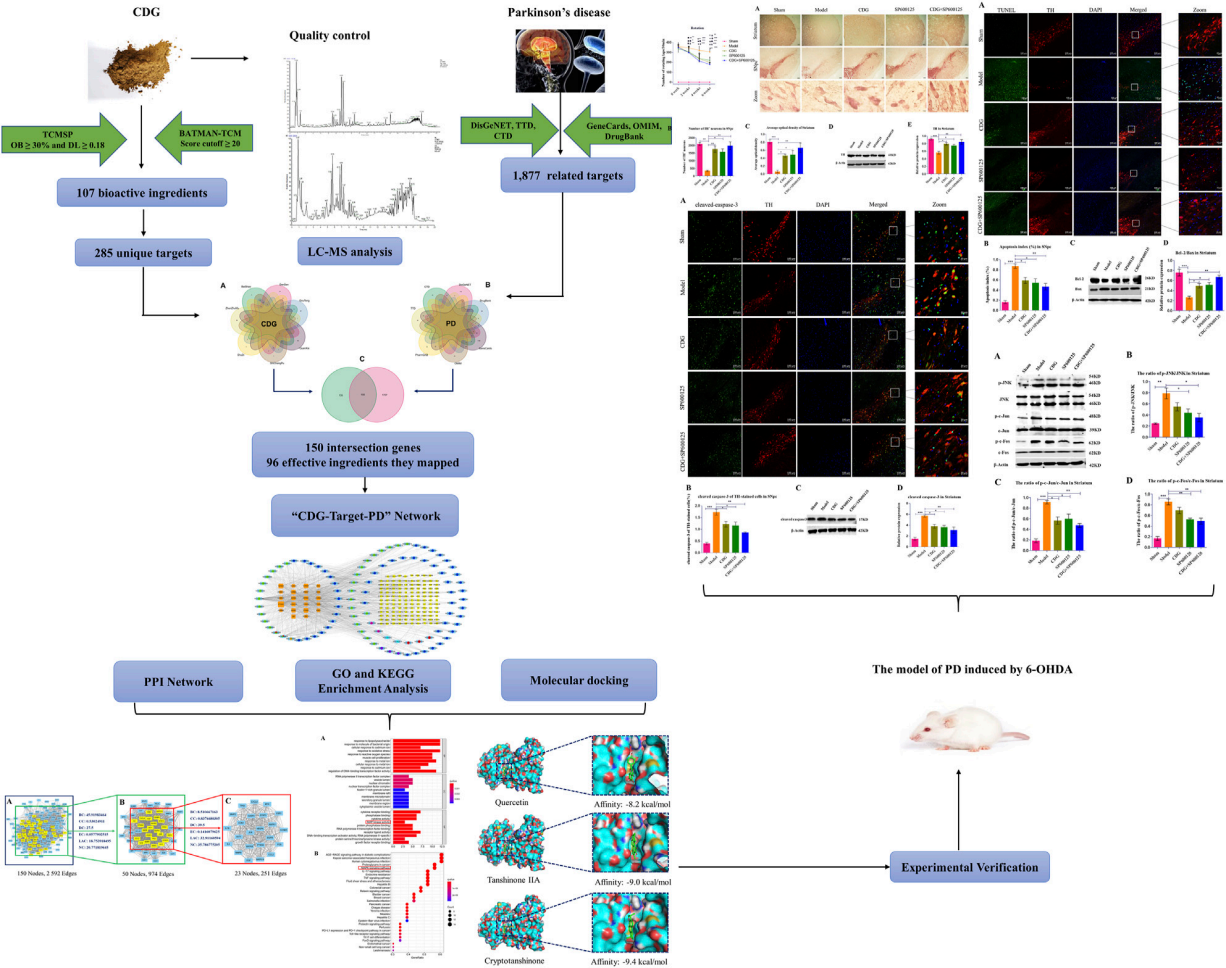
The graphical abstract of this study.

The graphical abstract of this study.

In the published article, there was an error in Figure 6A, Figure 7A, Figure 8C and the Graphical Abstract as published. The striatum of Figure 6A and the CDG + SP600125 group of Figure 7A in the article were not the final version. This may have been caused by an initial error in the image layout editing process. The β-Actin in Figure 8C is misused. Figure 6A, Figure 7A and Figure 8C are part of the Graphical Abstract, so the Graphical Abstract also needs to be corrected. The corrected Figure 6, Figure 7, Figure 8, Graphical Abstract and their captions appear below.

**FIGURE 6:**
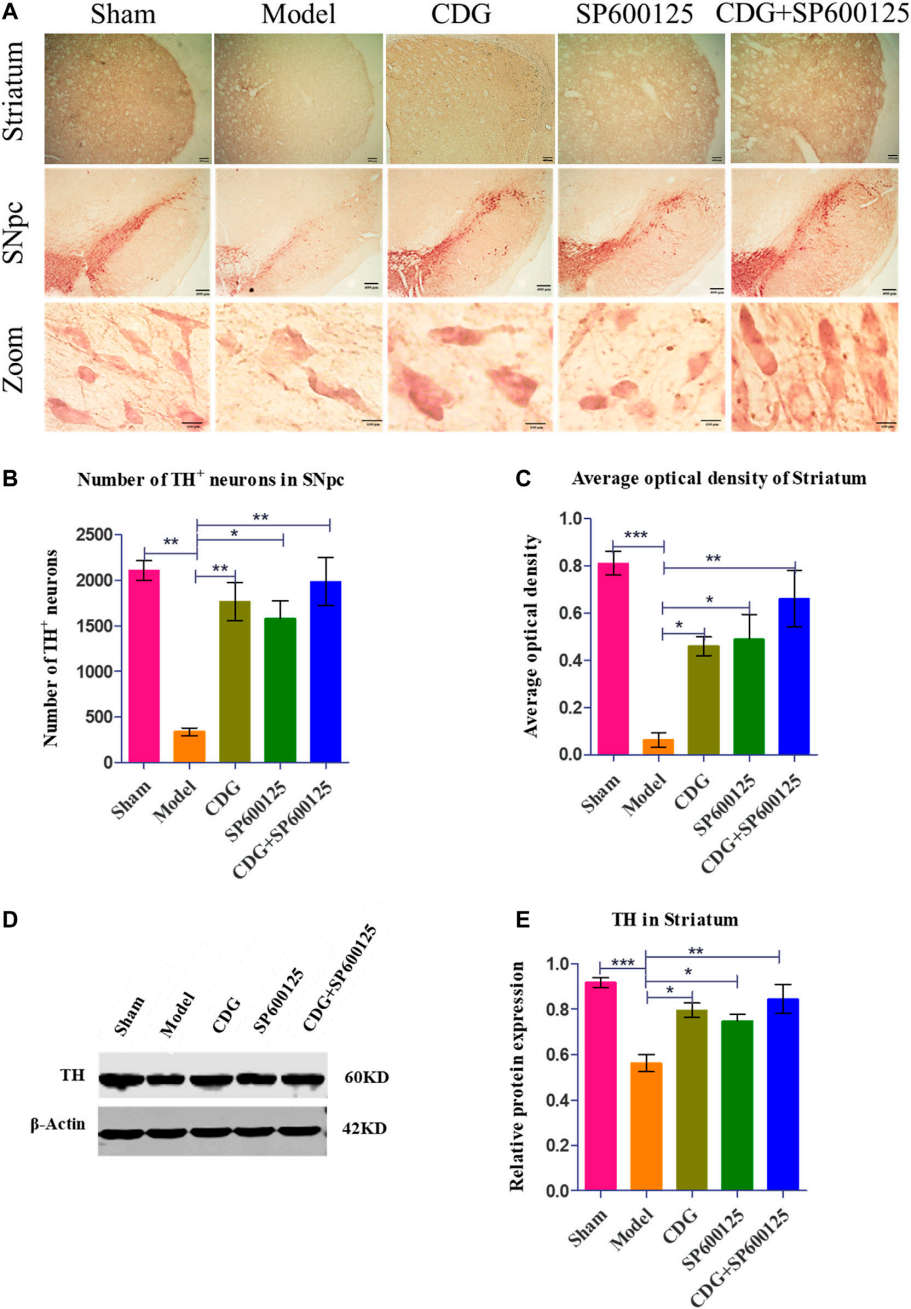
6-OHDA-induced loss of DA neurons in the nigrostriatal pathway of the rat brain. **(A)** DAB staining of TH on midbrain sections in each group (Scale bar: 400 μm; Zoomed Scale bar: 100 μm). **(B)** Stereological counts of TH-positive cells of the SNpc at 6 weeks after 6-OHDA intoxication. **(C)** Average optical density of striatum in each group. **(D)** The expression level of TH proteins was detected with Western Blot in the striatum. **(E)** The expression level of TH protein in each group. β-Actin served as control. Statistical analysis was performed with One-Way ANOVA, *n* = 3. Significant differences were indicated by *p < 0.05, **p < 0.01, ***p < 0.001.

**FIGURE 7:**
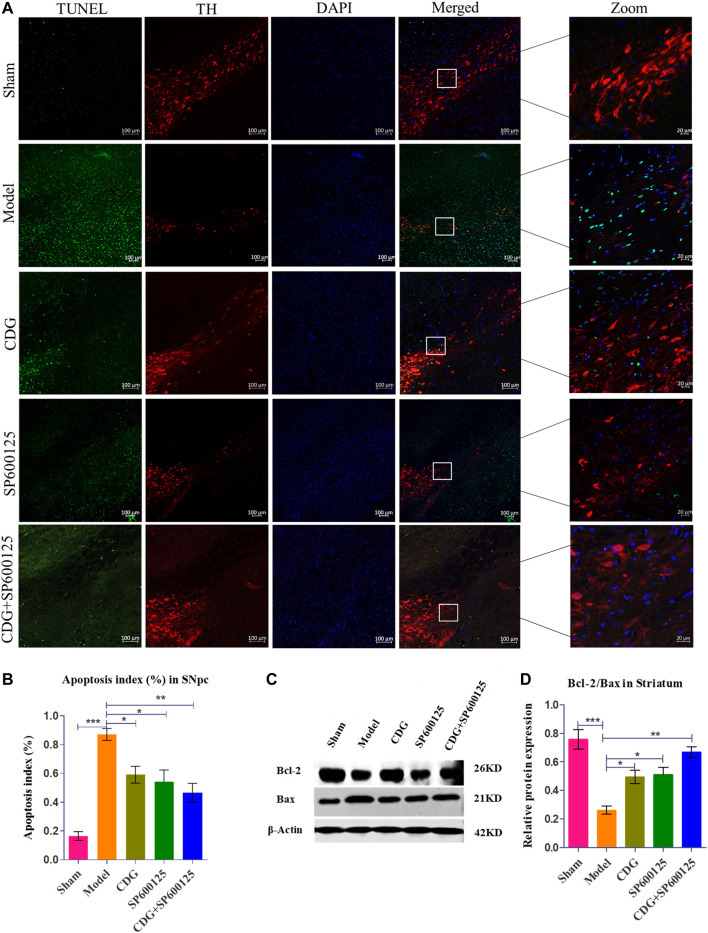
6-OHDA induced nigrostriatal pathway apoptosis of the PD rat brain. **(A)** TUNEL assay of apoptosis in each group. Representative confocal fluorescent images of the SNpc with TUNEL (green), TH (red), and DAPI (blue) (scale bar = 100 μm; Zoomed Scale bar; 20 μm). **(B)** Apoptosis index of the SNpc at 6 weeks after 6-OHDA intoxication. **(C,D)** The protein expression level of Bcl-2 and Bax were detected with Western Blot in the striatum. Statistical analysis was performed with One-Way ANOVA, *n* = 3. Significant differences were indicated by *p < 0.05, **p < 0.01, ***p < 0.001.

**FIGURE 8:**
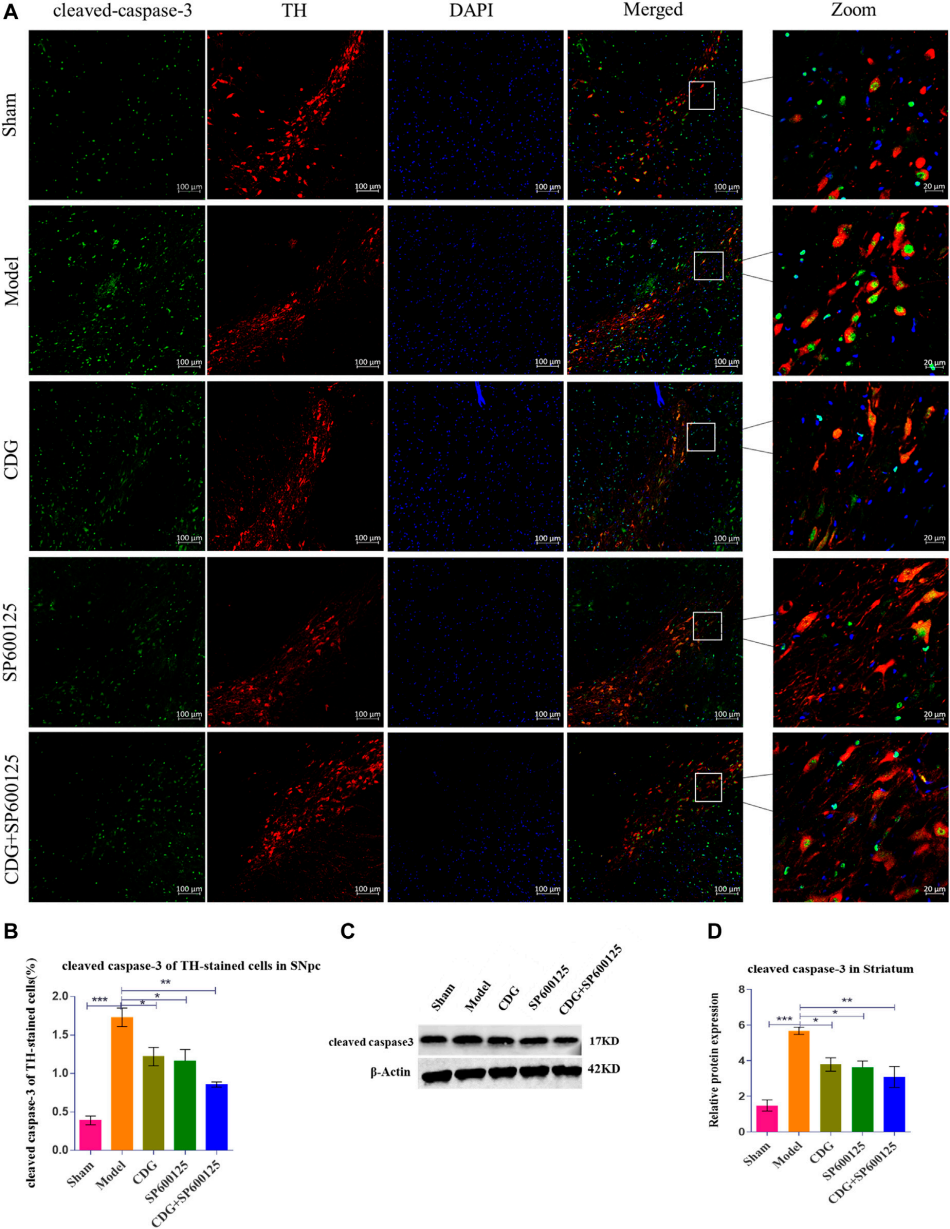
Expression of cleaved caspase-3 protein. **(A)** cleaved caspase-3 was detected by IF in each group. cleaved caspase-3 (green), TH (red) and DAPI (blue). (scale bar = 100 μm; Zoomed Scale bar: 20 μm). **(B)** The cleaved caspase-3 of TH-stained cells were calculated. **(C,D)** The expression level of cleaved caspase-3 protein was detected with Western Blot in the Striatum. β-Actin served as control. Statistical analysis was performed with One-Way ANOVA, Turkey’s multiple comparison test, *post hoc*, *n* = 3. Significant differences were indicated by ^*^ *p* < 0.05, ^**^ *p* < 0.01, ^***^ *p* < 0.001.

The authors apologize for this error and state that this does not change the scientific conclusions of the article in any way. The original article has been updated.

